# Nomogram for hospital-acquired venous thromboembolism among patients with cardiovascular diseases

**DOI:** 10.1186/s12959-024-00584-w

**Published:** 2024-01-30

**Authors:** Qin Luo, Xin Li, Zhihui Zhao, Qing Zhao, Zhihong Liu, Weixian Yang

**Affiliations:** 1 Center for Pulmonary Vascular Diseases, National Center for Cardiovascular Diseases, Fuwai Hospital, Chinese Academy of Medical Sciences and Peking Union Medical College, No.167 Beilishi Rd, Xicheng DistrictBeijing, 100037 China; 2 Department of Cardiology, National Center for Cardiovascular Diseases, Fuwai Hospital, Chinese Academy of Medical Sciences and Peking Union Medical College, Beijing, China

**Keywords:** Nomogram, Venous thromboembolism, Cardiovascular diseases, Padua score, Prediction model, Hospital-acquired

## Abstract

**Background:**

Identifying venous thromboembolism (VTE) is challenging for patients with cardiovascular diseases due to similar clinical presentation. Most hospital-acquired VTE events are preventable, whereas the implementation of VTE prophylaxis in clinical practice is far from sufficient. There is a lack of hospital-acquired VTE prediction models tailored specifically designed for patients with cardiovascular diseases. We aimed to develop a nomogram predicting hospital-acquired VTE specifically for patients with cardiovascular diseases.

**Material and methods:**

Consecutive patients with cardiovascular diseases admitted to internal medicine of Fuwai hospital between September 2020 and August 2021 were included. Univariable and multivariable logistic regression were applied to identify risk factors of hospital-acquired VTE. A nomogram was constructed according to multivariable logistic regression, and internally validated by bootstrapping.

**Results:**

A total of 27,235 patients were included. During a median hospitalization of four days, 154 (0.57%) patients developed hospital-acquired VTE. Multivariable logistic regression identified that female sex, age, infection, pulmonary hypertension, obstructive sleep apnea, acute coronary syndrome, cardiomyopathy, heart failure, immobility, central venous catheter, intra-aortic balloon pump and anticoagulation were independently associated with hospital-acquired VTE. The nomogram was constructed with high accuracy in both the training set and validation (concordance index 0.865 in the training set, and 0.864 in validation), which was further confirmed in calibration. Compared to Padua model, the Fuwai model demonstrated significantly better discrimination ability (area under curve 0.865 vs. 0.786, net reclassification index 0.052, 95% confidence interval 0.012–0.091, *P* = 0.009; integrated discrimination index 0.020, 95% confidence interval 0.001–0.039, *P* = 0.051).

**Conclusion:**

The incidence of hospital-acquired VTE in patients with cardiovascular diseases is relatively low. The nomogram exhibits high accuracy in predicting hospital-acquired VTE in patients with cardiovascular diseases.

**Supplementary Information:**

The online version contains supplementary material available at 10.1186/s12959-024-00584-w.

## Introduction

Venous thromboembolism (VTE) comprises deep venous thrombosis (DVT) and pulmonary embolism (PE), and is regarded as one of the major causes of mortality. Hospitalization is one of the major causes of VTE, and about 15% of high-risk patients can develop VTE during hospitalization [1]. Identifying VTE in patients with cardiovascular diseases is challenging due to the non-specific symptoms such as dyspnea, chest pain and syncope, which are also commonly observed in patients with cardiovascular diseases.

Most hospital-acquired VTE events are preventable, whereas the implementation of VTE prophylaxis in clinical practice is far from sufficient. Several VTE prediction models have been developed to identify hospitalized patients with increased risk of developing VTE, and promote adequate anticoagulation prophylaxis [2, 3]. However, most risk assessment tools do not take specific features of patients with cardiovascular diseases into consideration. The 9th American College of Chest Physicians Guidelines of preventing VTE in nonsurgical patients recommended Padua score for VTE prediction in medical patients [4], whereas the predictive ability of Padua score in medical patients varied significantly across different studies, ranging from 49.1% to 77.1% [5–7].

In the present study, we aim to investigate the incidence of hospital-acquired VTE, and develop a nomogram to facilitate clinicians identifying patients with cardiovascular diseases at high risk of developing hospital-acquired VTE.

## Material and methods

### Study design and population

This retrospective cohort was conducted in Fuwai hospital, National Center for Cardiovascular Diseases, Chinese Academy of Medical Sciences (Beijing, China), which is a cardiovascular specialized hospital with 35 wards, 1275 beds and over 700, 000 inpatients per year (https://www.fuwaihospital.org/Hospitals/Main?type=1). As a cardiovascular specific hospital, internal medicine department comprises coronary heart disease center, cardiac arrhythmia center, hypertension center, respiratory and pulmonary vascular diseases center, intensive care center, endocrinology center, dyslipidemia and cardiovascular disease center, heart failure care center, special care medical center, cardiomyopathy center and kidney center. The annual report of Fuwai hospital was attached in the supplementary materials (https://www.fuwaihospital.org/News/Columns/List/763), which further detailed the ward structure and setting of Fuwai hospital. The study was conducted according to the Declaration of Helsinki, and the Ethics Committee of Fuwai hospital approved the study protocol (Approval No. 2021–1543). Informed consent was waived due to the retrospective design. All consecutive patients admitted to internal medicine of Fuwai hospital between September, 2020 to August, 2021 were eligible. Exclusion criteria were: (1) patients’ length of hospitalization less than 3 days; (2) patients admitted primarily for VTE; (3) patients admitted to thrombosis center because patients in this department were commonly admitted for VTE; (4) identification of VTE within 48 h after admission, as the origin of the VTE is extrinsic to the hospital setting as opposed to being intrinsic; (5) patients without cardiovascular diseases. For recurrent admission, only the first admission was included into analysis.

The clinical data were collected via an electronic medical record system and diagnosis of diseases was captured by International Classification of Diseases (ICD) coding and keywords extraction methods from discharge diagnosis. The discharge diagnosis consisted of the primary admitting diagnosis and all comorbidities. We compared the results of ICD with keywords extraction. Any discrepancy would be checked by the third reviewer manually. We also conducted random spot checks on 30% of all patients manually. When investigating the risk factors for hospital-acquired VTE, we included interventional and medical treatments administered prior to the occurrence of VTE, whereas treatments after VTE events were not taken into account. Anticoagulation treatment included low molecular weight heparin, low dose unfractionated heparin, fondaparinux, direct oral anticoagulation agents and warfarin.

### Cardiovascular diseases

Cardiovascular diseases included but were not limited to coronary heart disease, hypertension, cardiomyopathy, heart failure, pulmonary hypertension, congenital heart disease, atrial fibrillation, and the definitions of diseases were provided in the supplementary methods.

### Outcome

The primary outcome of this study was hospital-acquired VTE, which was defined as a new occurrence of VTE after hospitalization for over 48 h [8]. Patients were followed up until hospital discharge. Diagnosis of DVT was confirmed by compression ultrasonography. Diagnosis of PE was confirmed by computed tomography pulmonary angiography or ventilation/perfusion single-photon emission computed tomography. ICD codes used to identify outcome events were detailed in the Supplementary Table S1.

### Statistical analysis

Distribution of variables was examined by Kolmogorov-Smirnova test. Accordingly, normally distributed continuous parameters were examined by independent-sample *t* tests and presented as the mean ± standard deviation, abnormally distributed continuous parameters were examined by Mann–Whitney *U* tests and presented as the median (interquartile range). Categorical variables were given as counts (percentages), and the *Chi*-square tests were used to compare difference between groups. Univariable logistic regressions were used to identify risk factors associated with occurrence of VTE. Variables reaching statistical significance (*P* < 0.05) were included into multivariable logistic regressions (forward stepwise methods). To avoid overfitting, the number of events per predictor was set as 10 [9]. A nomogram for predicting VTE was constructed according to multivariable logistic regression. Receiver operator characteristic curve (ROC) analyses and area under curve (AUC) were applied to evaluate predictive ability of models. Net reclassification index and integrated discrimination index were used to compare clinical usefulness and net benefit of both models. Decision curve analysis was employed to evaluate the performance of the models. Calibration curves were employed to assess and compare the precision of the models. Bootstrap method was used for internal validation with 20,000 resamples. In the bootstrapping process, the entire dataset serves as the basis for both the original database and subsequent validation datasets. The validation dataset is constructed by drawing random samples with replacement from the original dataset. Not a single missing value was replaced. A two-tailed *P* value less than 0.05 was considered of statistical significance. R (version 4.3.1, R Foundation for Statistical Computing, Vienna, Austria) was used for statistical analyses.

## Results

### Patients’ enrollment

**Fig. 1:**
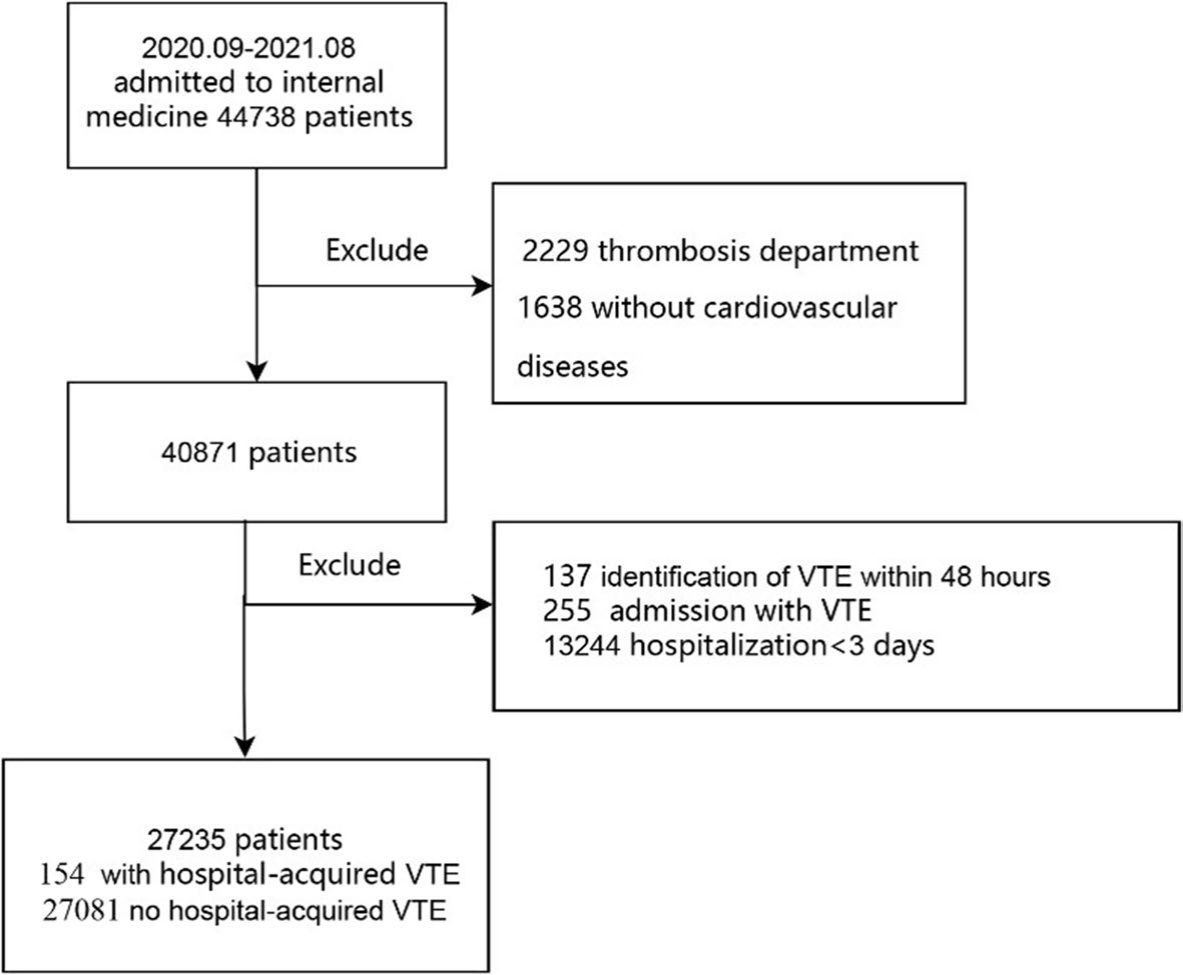
Flow diagram of participant enrollment and exclusion. VTE, venous thromboembolism

From September, 2020 to August, 2021, 44,738 patients were admitted to internal medicine department of Fuwai hospital (Fig. 1). Among them, 1638 patients were excluded due to no history of cardiovascular diseases, 2229 patients due to admission to the thrombosis department, 255 patients due to admission with VTE, 137 patients due to identification of VTE within 48 h after admission, 13,244 patients due to hospitalization less than 3 days. Finally, 27,235 patients were included. Among the cohort, 8450 individuals (31.03%) were female, with a median age of 60 (51, 67) years. Coronary heart disease (17,626, 64.72%) and hypertension (16,879, 61.98%) were the most commonly observed cardiovascular diseases, whereas heart failure (1337, 4.91%) and pulmonary hypertension (974, 3.58%) were the least common (Supplementary Table S2).

During a median hospitalization of four days, 154 patients developed hospital-acquired VTE, whereas 27,081 patients did not. Among patients who developed hospital-acquired VTE, 92 patients (59.74%) had DVT, 32 patients (20.78%) had PE, and 30 patients (19.48%) had both PE and DVT.

### Incidence and distribution of hospital-acquired VTE

The incidence of hospital-acquired VTE in the total cohort was 0.57%. Of note, the ICU had the highest incidence of hospital-acquired VTE (7.62%). On the contrary, department of coronary heart disease had the lowest incidence of VTE (0.09%) despite having the highest number of admitted patients.

### Comparison of patients with and without hospital-acquired VTE

Compared to patients without hospital-acquired VTE, patients experiencing hospital-acquired VTE were significantly older (67.17 ± 13.71 years vs. 58.41 ± 13.21 years, *P* < 0.001), had worse New York Heart Association function class (NYHA FC) (III/ IV 40.91% vs. 6.47%, *P* < 0.001) and higher D-dimer [1.95 (1.12, 3.56) ug/ml vs. 0.79 (0.61, 1.26) ug/ml, *P* < 0.001] (Table 1). Severe cardiac diseases were also more prevalent in patients with hospital-acquired VTE, including acute coronary syndrome, heart failure and cardiomyopathy. Besides, these patients also had more comorbidities, including respiratory failure, infection, renal insufficiency, pulmonary hypertension, obstructive sleep apnea and hepatic insufficiency.

**Table 1:** Demographics of patients with/ without hospital-acquired VTE

Variable	Without hospital-acquired VTE (*n* = 27,081)	With hospital-acquired VTE (*n* = 154)	*P*-value
Female, n (%) (*n* = 27,235)	8372 (30.9)	78 (50.6)	** < 0.001**
Age, years (*n* = 27,235)	58.41 ± 13.21	67.17 ± 13.71	** < 0.001**
BMI, kg/m^2^ (*n* = 27,129)	25.63 ± 3.51	25.30 ± 3.62	0.282
D-Dimer, ug/ml (*n* = 5896)	0.79 (0.61, 1.26)	1.95 (1.12, 3.56)	** < 0.001**
Prior venous thromboembolism, n (%) (*n* = 27,129)	10 (0.0)	3 (1.9)	** < 0.001**
Duration of hospitalization, days (*n* = 27,235)	4 (3, 6)	12 (8, 17)	** < 0.001**
Time to VTE, days (*n* = 27,235)	NA	5 (4, 8)	
NYHA FC (*n* = 9372)			** < 0.001**
I, n (%)	4106 (15.16)	16 (10.39)	
II, n (%)	3407 (12.58)	28 (18.18)	
III, n (%)	1231 (4.55)	32 (20.78)	
IV, n (%)	521 (1.92)	31 (20.13)	
Ward (*n* = 27,235)			** < 0.001**
ICU, n (%)	2655 (9.8)	93 (60.4)	
Other, n (%)	24,426 (90.2)	61 (39.6)	
Coronary heart disease (*n* = 27,235)			** < 0.001**
Chronic coronary syndrome, n (%)	14,829 (54.8)	55 (35.7)	
Acute coronary syndrome, n (%)	2710 (10.0)	32 (20.8)	
Hypertension, n (%) (*n* = 27,235)	16,788 (62.0)	91 (59.1)	0.460
Atrial fibrillation, n (%) (*n* = 27,235)	4734 (17.5)	44 (28.6)	** < 0.001**
Cardiomyopathy, n (%) (*n* = 27,235)	1919 (7.1)	38 (24.7)	** < 0.001**
Heart failure, n (%) (*n* = 27,235)	5212 (19.2)	91 (59.1)	** < 0.001**
Pulmonary hypertension, n (%) (*n* = 27,235)	930 (3.4)	44 (28.6)	** < 0.001**
Congenital heart diseases, n (%) (*n* = 27,235)	338 (1.2)	2 (1.3)	0.955
Valvular heart disease, n (%) (*n* = 27,235)	3077 (11.4)	66 (42.9)	** < 0.001**
Infection, n (%) (*n* = 27,235)	1595 (5.9)	49 (31.8)	** < 0.001**
Renal insufficiency, n (%) (*n* = 27,235)	1199 (4.4)	34 (22.1)	** < 0.001**
Obstructive sleep apnea, n (%) (*n* = 27,235)	1940 (7.2)	23 (14.9)	** < 0.001**
Stroke, n (%) (*n* = 27,235)	2458 (9.1)	17 (11.0)	0.398
Respiratory failure, n (%) (*n* = 27,235)	115 (0.4)	11 (7.1)	** < 0.001**
Hyperhomocysteinemia, n (%) (*n* = 27,235)	740 (2.7)	5 (3.2)	0.887
Rheumatic disease, n (%) (*n* = 27,235)	117 (0.4)	1 (0.6)	0.489
Diabetes, n (%) (*n* = 27,235)	8307 (30.7)	53 (34.4)	0.316
Malignancy, n (%) (*n* = 27,235)	376 (1.4)	5 (3.2)	0.107
Arthritis, n (%) (*n* = 27,235)	173 (0.6)	1 (0.6)	0.987
Varicose vein, n (%) (*n* = 27,235)	238 (0.9)	5 (0.6)	0.101
Hepatic insufficiency, n (%) (*n* = 27,235)	1739 (6.4)	28 (18.2)	** < 0.001**
Recent trauma or surgery, n (%) (*n* = 27,235)	8 (0.0)	0 (0.0)	1.000
Thrombophilia, n (%) (*n* = 27,235)	1 (0.0)	0 (0.0)	1.000
Invasive procedure^a^ (*n* = 27,235)			
Central venous catheter, n (%)	153 (0.6)	14 (9.1)	** < 0.001**
IABP, n (%)	90 (0.3)	10 (6.5)	** < 0.001**
ECMO, n (%)	4 (0.0)	0 (0.0)	1.000
Pacemaker, n (%)	944 (3.5)	9 (5.8)	0.112
Stent implantation, n (%)	64 (0.2)	0 (0.0)	0.546
Atrial/ventricular septal defect occlusion, n (%)	30 (0.1)	0 (0.0)	0.679
Left atrial appendage occlusion, n (%)	13 (0.0)	0 (0.0)	0.786
Defibrillators implantation, n (%)	61 (0.2)	0 (0.0)	0.555
Right heart catheter, n (%)	86 (0.3)	0 (0.0)	0.484
Mechanical ventilation, n (%) (*n* = 27,235)	691 (2.6)	12 (7.8)	** < 0.001**
Immobility, n (%) (*n* = 27,235)	48 (0.2)	12 (7.8)	** < 0.001**
Medical treatment^a^ (*n* = 27,235)			
Steroids, n (%)	389 (1.4)	3 (1.9)	0.847
Anticoagulation, n (%)	22,363 (82.6)	108 (70.1)	** < 0.001**
Antiplatelet, n (%)	19,971 (73.7)	86 (55.8)	** < 0.001**
Statin, n (%)	20,876 (77.1)	104 (67.5)	**0.005**
Padua score^b^ (*n* = 27,235)			** < 0.001**
Low risk, n (%)	26,663 (98.5)	129 (83.8)	
High risk, n (%)	418 (1.5)	25 (16.2)	

*BMI* Body mass index, *ECMO* Extracorporeal membrane oxygenation, *IABP* Intra-aortic balloon pump, *ICU* Intensive care unit, *NYHA FC* New York Heart Failure Association Functional Classification, *VTE* Venous thromboembolism. ^a^Invasive procedure and medical treatment prior to VTE were included, whereas those after VTE events were not taken into account. ^b^Low risk is defined as Padua score < 4 points, high risk is defined as Padua score ≥ 4 points, in line with 9th American College of Chest Physicians Evidence-Based Clinical Practice Guidelines (https://doi.org/10.1378/chest.11-2296.)

The duration of hospitalization was also significantly longer in patients who developed VTE [12 (8, 17) days vs. 4 (3, 6) days, *P* < 0.001], and the median time of identifying hospital-acquired VTE was the fifth day after admission. During the hospitalization, patients who developed VTE were more likely to undergo invasive procedures, including central venous catheterization (9.1% vs. 0.6%, *P* < 0.001), intra-aortic balloon pump (IABP) (6.5% vs. 0.3%, *P* < 0.001) and mechanical ventilation (7.8% vs. 2.6%, *P* < 0.001). Moreover, these patients had higher proportion of immobility (7.8% vs. 0.2%, *P* < 0.001). During the hospitalization period, patients who developed VTE were less likely to receive anticoagulants (70.1% vs. 82.6%, *P* < 0.001), antiplatelet agents (55.8% vs. 73.7%, *P* < 0.001) and statin (67.5% vs. 77.1%, *P* = 0.005).

### Factors associated with hospital-acquired VTE

**Fig. 2:**
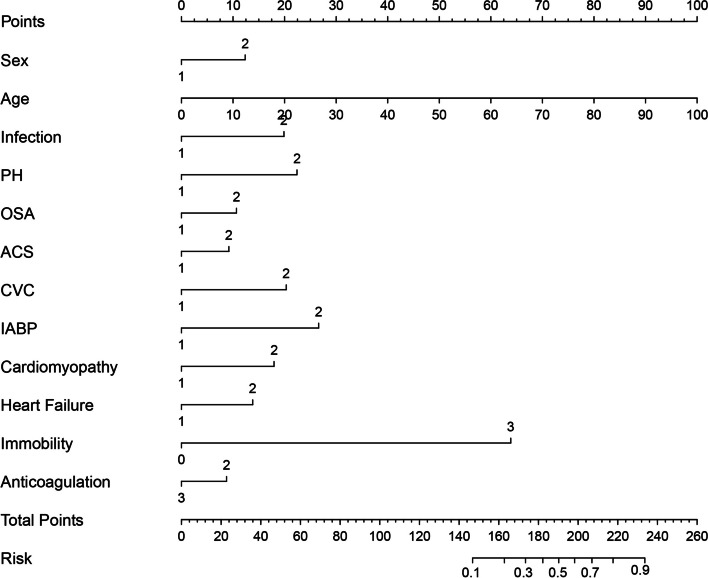
Nomogram for predicting hospital-acquired VTE in patients with cardiovascular diseases. ACS, acute cardiovascular syndrome; CMP, cardiomyopathy; CVC, central venous catheterization; IABP, intra-aortic balloon pump; PH, pulmonary hypertension; VTE, venous thromboembolism. In order to calculate the probability of hospital-acquired VTE, draw a vertical line straight upward from the predictor to the points axis to obtain the value, then sum the points of each predictor, this sum on the total points axis of the nomogram corresponded with the probability of hospital-acquired VTE, which can be determined by drawing a vertical line downward. For example, for a 60-year old female patient who had received central venous catheterization and was immobile during hospitalization without other risk factors and did not receive anticoagulants, the total points (156) = female sex (12 points) + 60 years old (60 points) + immobility (64 points) + central venous catheterization (20 points), and the probability of VTE was about 15%

**Fig. 3:**
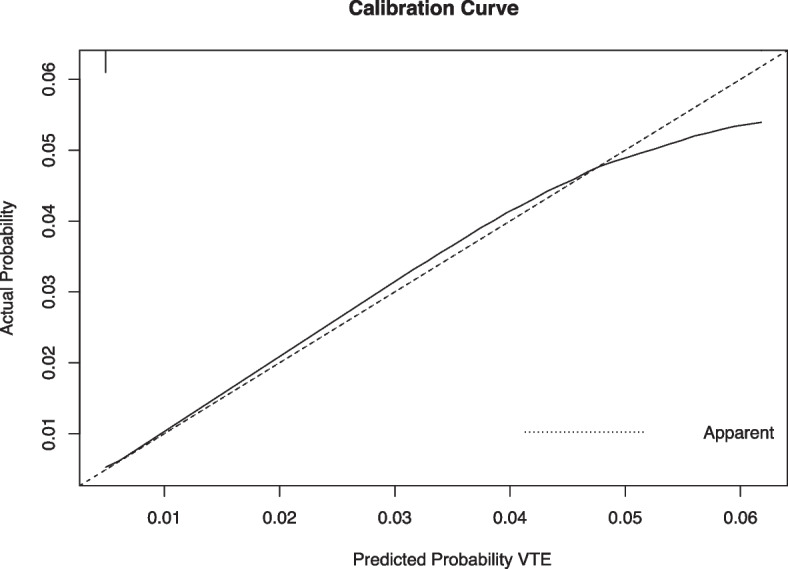
Calibration curve of nomogram. Calibration curve indicated high accuracy of nomogram in predicting VTE

Univariable logistic regression identified that female, age, D-dimer, NYHA FC, prior VTE, infection, renal insufficiency, pulmonary hypertension, obstructive sleep apnea, valvular heart disease, chronic coronary syndrome, acute coronary syndrome, cardiomyopathy, respiratory failure, heart failure, hepatic insufficiency, immobility, atrial fibrillation, mechanical ventilation, central venous catheterization and IABP were associated with increased risk of developing hospital-acquired VTE, whereas administration of anticoagulants, antiplatelet and statin was associated with reduced risk of hospital-acquired VTE (Table 2). Multivariable logistic regression further identified that female sex, age, infection, pulmonary hypertension, obstructive sleep apnea, acute coronary syndrome, cardiomyopathy, heart failure, immobility, central venous catheter, IABP and anticoagulation were independently associated with hospital-acquired VTE (Table 3 and Supplementary Table S3). Nomogram was constructed based on multivariable logistic regression (Fig. 2). The higher the total points, the higher the risk of developing VTE. The predictive ability of novel Fuwai nomogram was high (concordance index 0.865), which was further confirmed by internal validation (bias adjusted concordance index 0.864) and calibration (Fig. 3).

**Table 2:** Univariable logistic analysis of risk for hospital-acquired VTE

Variable	OR	95% CI	*P*-value
Female	2.294	1.671–3.149	** < 0.001**
Age	1.065	1.049–1.081	** < 0.001**
BMI	0.974	0.928–1.022	0.282
D-Dimer	1.192	1.148–1.238	** < 0.001**
NYHA FC	2.574	2.136–3.101	** < 0.001**
Prior venous thromboembolism	7.334	3.828–14.049	** < 0.001**
Hypertension	0.941	0.801–1.106	0.460
Infection	7.457	5.292–10.506	** < 0.001**
Renal insufficiency	6.116	4.161–8.990	** < 0.001**
Pulmonary hypertension	11.248	7.881–16.052	** < 0.001**
Obstructive sleep apnea	2.275	1.457–3.552	** < 0.001**
Stroke	1.243	0.750–2.061	0.399
Valvular heart disease	5.851	4.243–8.068	** < 0.001**
Chronic coronary syndrome	0.528	0.369–0.755	** < 0.001**
Acute coronary syndrome	1.682	1.101–2.568	**0.016**
Cardiomyopathy	4.295	2.969–6.214	** < 0.001**
Respiratory failure	18.037	9.511–34.209	** < 0.001**
Hyperhomocysteinemia	1.194	0.489–2.921	0.697
Rheumatic disease	1.506	0.209–10.852	0.684
Diabetes	1.186	0.850–1.655	0.316
Heart failure	6.061	4.389–8.369	** < 0.001**
Malignant tumor	2.383	0.972–5.844	0.058
Hepatic insufficiency	3.238	2.144–4.891	** < 0.001**
Immobility	47.593	24.754–91.506	** < 0.001**
Atrial fibrillation	1.888	1.329–2.682	** < 0.001**
Mechanical ventilation	3.227	1.782–5.846	** < 0.001**
Central venous catheterization	17.600	9.934–31.181	** < 0.001**
IABP	20.826	10.619–40.844	** < 0.001**
Steroids	1.363	0.433–4.293	0.597
Anticoagulation	0.495	0.350–0.700	** < 0.001**
Antiplatelet	0.450	0.327–0.620	** < 0.001**
Statin	0.618	0.441–0.867	**0.005**
Padua Score (continuous)	2.103	1.922–2.301	** < 0.001**
Padua Score (category)	12.362	7.969–19.176	** < 0.001**

*BMI* Body mass index, *CI* Confidence interval, *IABP* Intra-aortic balloon pump, *ICU* Intensive care unit, *NYHA FC* New York Heart Failure Association Functional Classification, *OR* Odds ratio, *VTE* Venous thromboembolism

**Table 3:** Multivariable logistic analysis of risk for hospital-acquired VTE

Variable	OR	95% CI	*P*-value
Female	1.869	1.337–2.612	** < 0.001**
Age	1.052	1.037–1.067	** < 0.001**
Infection	2.734	1.839–4.066	** < 0.001**
Pulmonary hypertension	3.107	2.030–4.755	** < 0.001**
OSA	1.716	1.054–2.792	**0.030**
Acute coronary syndrome	1.592	1.001–2.532	**0.049**
Cardiomyopathy	2.481	1.560–3.943	** < 0.001**
Heart failure	2.012	1.335–3.032	**0.001**
Immobility	2.936	2.277–3.786	** < 0.001**
Central venous catheter	2.796	1.328–5.885	**0.007**
IABP	3.844	1.543–9.578	**0.004**
Anticoagulation	0.642	0.438–0.942	**0.023**

Variables were entered with forward stepwise (likelihood ratio)

*CI* Confidence interval, *IABP* Intra-aortic balloon pump, *OSA* Obstructive sleep apnea, *OR* Odds ratio, *VTE* Venous thromboembolism

### Comparison of VTE risk prediction models

**Fig. 4:**
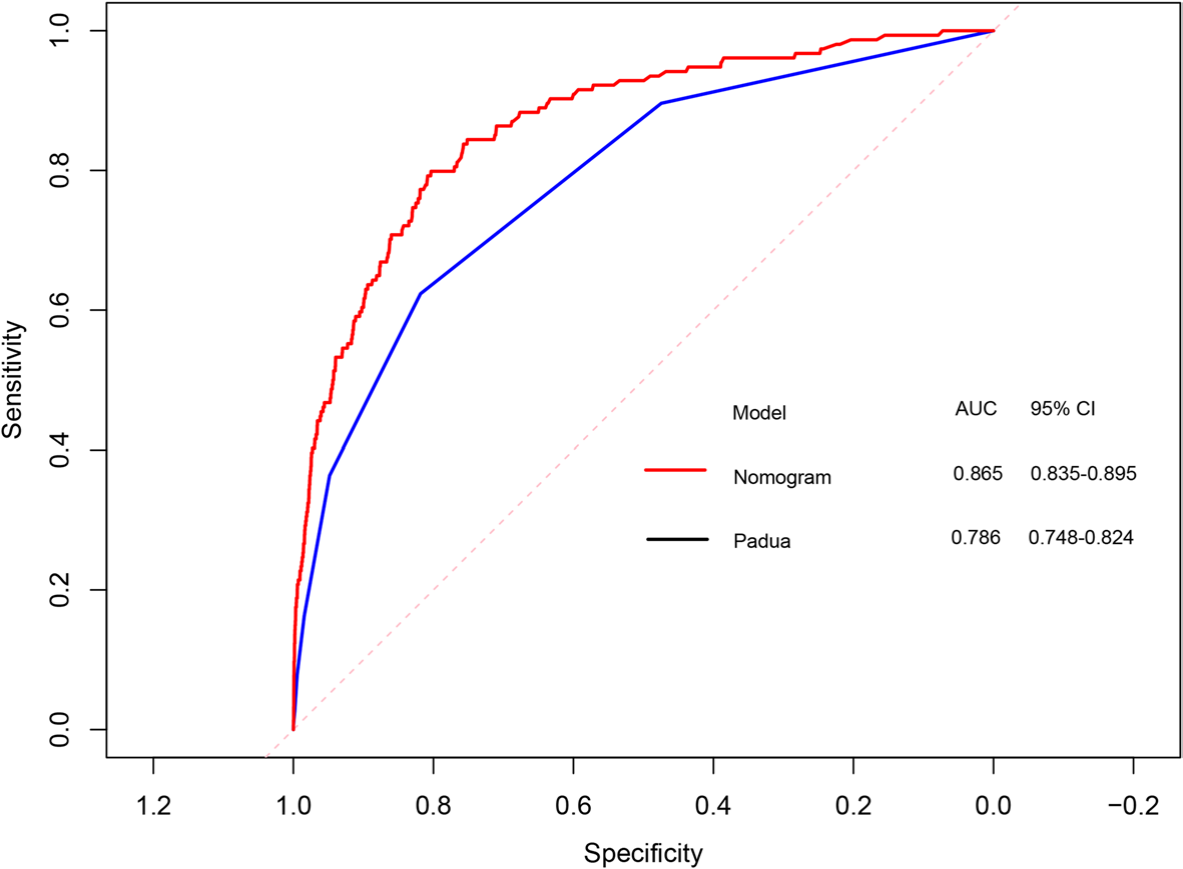
Receiver operator characteristic curve of Padua score and Fuwai nomogram. AUC, area under curve; 95% CI, 95% confidence interval

**Fig. 5:**
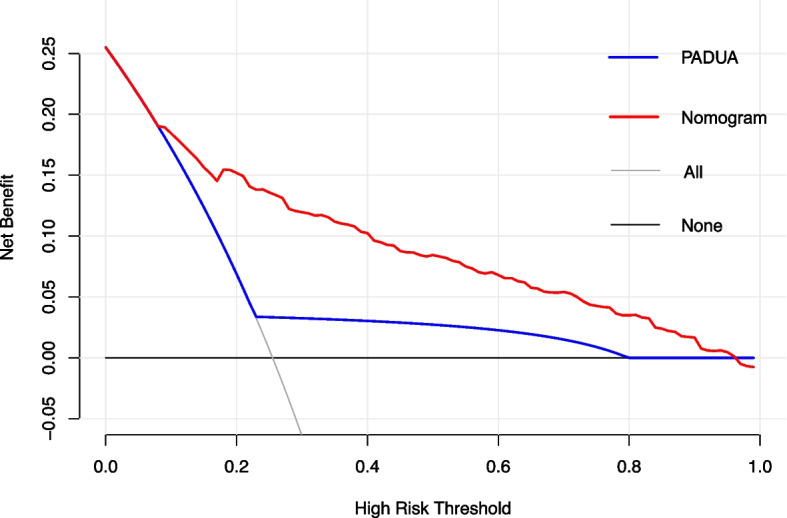
Decision curve analysis of Padua score and Fuwai nomogram. In the context of decision curve analysis, the x-axis illustrates threshold values for risks of VTE, whereas the y-axis denotes the net benefit for different cutoffs of primary endpoint risk. The greater the deviation of prediction models from the grey line and the horizontal black line, the greater the corresponding net benefit. The Fuwai nomogram demonstrated the larger net benefit across the range of risk of hospital-acquired VTE compared with Padua score. As the guideline suggested, Padua score was categorized two categories. Low risk is defined as Padua score < 4 points, high risk is defined as Padua score ≥ 4 points, in line with 9th American College of Chest Physicians Evidence-Based Clinical Practice Guidelines [4]

The predictive ability of Padua model (AUC 0.786, 95% CI 0.748–0.824, sensitivity 62.34%, specificity 81.84%) (Fig. 4) was lower than that of nomogram (AUC 0.865, 95% CI 0.835–0.895, sensitivity 79.87%, specificity 80.34%) (Fig. 3). Compared with Padua model, the nomogram had significantly better discrimination ability (net reclassification index 0.052, 95% CI 0.012–0.091, *P* = 0.009; integrated discrimination index 0.020, 95% CI 0.001–0.039, *P* = 0.051). Decision Curve Analysis demonstrated that the nomogram had better performance of risk prediction than Padua score (Fig. 5).

## Discussion

This study reported the incidence of hospital-acquired VTE in medical patients with cardiovascular diseases and developed a risk prediction model for this specific population. The Fuwai nomogram has high accuracy in prediction of VTE in patients with cardiovascular diseases, which was further confirmed in validation.

### Incidence of hospital-acquired VTE

The incidence of hospital-acquired VTE in patients with cardiovascular diseases was 0.57%, which was relatively low compared with other studies [10–12]. Previous studies reported that the incidence of hospital-acquired VTE in medical patients ranging from 0.43% to 9.7% [3, 12, 13], and the highest incidence was observed in acutely ill hospitalized elderly patients. The discrepancy in incidence could be attributable to difference in study population. Patients with cardiovascular diseases were more commonly receiving anticoagulants, antiplatelets or statin, which could reduce the risk of VTE. Different observation period also accounts for the discrepancy. The observation period of previous studies was commonly 90 days after admission, whereas the current study only observed from admission to discharge.

Incidence of VTE also varied significantly in different departments, and the highest incidence was observed in ICU, which was consistent with previous studies [14]. Patients who were admitted to ICU often had more advanced diseases and stayed immobile, contributing to higher incidence of VTE.

### Factors associated with VTE

Previous studies had reported that acute coronary syndrome, heart failure, immobility, prior VTE, infection and renal insufficiency were associated with increased risk of developing VTE, which were also confirmed in the current study [15]. Besides well-validated risk factors, current study also identified specific risk factors of VTE in patients with cardiovascular diseases, including pulmonary hypertension and cardiomyopathy. Patients with pulmonary hypertension, especially chronic thromboembolic pulmonary hypertension, are predisposed to experience recurrent DVT due to venous congestion and sedentary lifestyle, and the incidence of VTE was as high as 1.69/100 person-year [16]. Similarly, patients with cardiomyopathy also have stagnant systemic circulation secondary to impaired cardiac contractility and are susceptible to developing VTE.

Besides individual intrinsic risk factors, extrinsic factors associated with clinical situation and procedures also contribute to development of VTE, including immobilization, ICU stay, central venous catheterization and mechanical ventilation, consistent with previous studies [17, 18]. Of note, we identified that IABP was a strong risk factor of VTE. IABP could lead to lower limb blood stagnation, and patients who underwent IABP are commonly confined to the bed, contributing to the development of VTE.

In the current study, 82.51% patients received anticoagulants, including low molecular weight heparin, low dose unfractionated heparin, fondaparinux, direct oral anticoagulation agents and warfarin. Several factors contribute to administration of anticoagulants, including mechanical valve, atrial fibrillation, interventional procedure. Although the anticoagulants might not be initially prescribed for VTE prophylaxis, which could also reduce the risk of VTE. Besides anticoagulation, we found that antiplatelet agents and statin could also protect patients from hospital-acquired VTE. Platelet activation also plays a role in the pathogenesis of venous thrombosis [19, 20]. Statin, which is primarily applied for reducing cholesterol, has also been demonstrated to be effective to reduce the risk of VTE in several studies. Statins are reported to reduce risk of VTE by 39% to 55% [21, 22]. The effect of statins to reduce thrombosis could be attributable to reduced expression of tissue factor and thrombin generation [21]. However, the 9th American College of Chest Physicians Guidelines of preventing VTE in nonsurgical patients did not recommend statins for routine VTE prevention given limited evidence and availability of well-established anticoagulants [4].

### Comparison with Padua

Although the guidelines recommended Padua score to predict risk of VTE in medical patients [4], the predictive ability of Padua score varied significantly in different studies. Zhou, H. et al. found that the Padua score could only identify 49.1% hospital-acquired VTE in medical patients [5]. In this study, we found that Padua score could predict 78.6% hospital-acquired VTE in patients with cardiovascular diseases, while the nomogram could predict 86.5% of VTE events. Several factors contribute to the discrepancy in predictive ability of both models. Padua score was derived from a small sample size study in medical patients. Invasive procedure was not included in Padua score, which is commonly performed in patients with cardiovascular diseases and presents as a strong risk factor of developing VTE. On the contrary, the Fuwai nomogram incorporated invasive procedures and specific cardiovascular diseases, which demonstrated high accuracy in predicting hospital-acquired VTE in patients with cardiovascular diseases. Difference in study population also accounts for the discrepancy. Padua recruited all medical patients, while the current study was based on patients who had cardiovascular diseases, and these patients were more likely to receive anticoagulants or antiplatelet treatment.

### Limitation

The current study has several limitations. Firstly, the current study is based on a single center. However, Fuwai hospital is the national cardiovascular center of China and our patients are from all over the country. Moreover, the current study has the largest sample size in terms of cardiovascular diseases. Therefore, the current study is representative of patients with cardiovascular diseases. Secondly, the observation period is relative short in the current study and some patients might experience VTE within 3 months after discharge, which warrants future studies with longer observation period. Thirdly, external validation was not performed in the current study due to unavailability of data. However, internal validation was performed to validate the current model, and the predictive value remained stable in the internal validation. Fourthly, it is challenging to discriminate therapeutic anticoagulation from prophylaxis anticoagulation in this study because many patients with cardiovascular diseases had indication for anticoagulation such as atrial fibrillation. The dosing and interval of anticoagulant treatment were not quantified due to significant heterogeneity in anticoagulants among patients. Fifthly, the data on bleeding events were not available due to the retrospective study design, which warrants future well-designed prospective studies. Sixthly, the incidence of VTE was reported to be lower in Asian than other ethnicities [23]. Moreover, the risk profiles of VTE also varied between Asian and other ethnicities, which might limit the generalizability of the finding in this study to other ethnicities.

## Conclusion

The incidence of hospital-acquired VTE in medical patients with cardiovascular diseases is relatively low. The Fuwai nomogram has high accuracy in prediction of hospital-acquired VTE in patients with cardiovascular diseases.

### Supplementary Information

**Additional file 1. **Supplementary methods. **Table S1.** ICD code of outcome events. **Table S2.** Number of patients with cardiovascular diseases. **Table S3.** Multivariable logistic analysis of risks for hospital-acquired VTE.

## Data Availability

All data generated or analyzed during this study are included in this manuscript.
