# Supplementary material for: Nomogram for hospital-acquired venous thromboembolism among patients with cardiovascular diseases
Source: Thromb J. 2024 Jan 30;22:15. doi: 10.1186/s12959-024-00584-w (PMC10826242; doi:10.1186/s12959-024-00584-w)
Supplement: Supplementary file 1 — Additional file 1. Supplementary methods. Table S1. ICD code of outcome events. Table S2. Number of patients with cardiovascular diseases. Table S3. Multivariable logistic analysis of risks for hospital-acquired VTE. [file 12959_2024_584_MOESM1_ESM.docx]

**Supplementary methods**

Definition of diseases

**Cardiomyopathy** is defined as diseases of the heart muscle, where the walls of the heart chambers have become stretched, thickened or stiff. This affects the heart's ability to pump blood around the body, including dilated, hypertrophic and restrictive cardiomyopathy^1^.

**Pulmonary hypertension** is defined as mean pulmonary arterial pressure >20mmHg, in line with 2022 ESC guideline^2^.

**Heart failure** is defined as is a clinical syndrome characterized by typical symptoms (e.g. breathlessness, ankle swelling and fatigue) that may be accompanied by signs (e.g. elevated jugular venous pressure, pulmonary crackles and peripheral oedema) caused by a structural and/or functional cardiac abnormality, resulting in a reduced cardiac output and/or elevated intracardiac pressures at rest or during stress^3^.

**Immobility** was defined as confinement on a bed for >24 hours, in line with previous studies^4^.

**Coronary heart disease** was defined as a type of heart disease where the arteries of the heart cannot deliver enough oxygen-rich blood to the heart**^5^.**

**Hypertension** was defined as hypertension is defined as office systolic blood pressure values at least 140 mmHg and/or diastolic blood pressure values at least 90 mmHg^6^.

**Congenital heart disease** was defined as a range of birth defects that affect the normal way the heart works^7^.

**Atrial fibrillation** was defined as a supraventricular tachyarrhythmia with uncoordinated atrial activation and consequently ineffective atrial contraction^8^.

**Reference**

1. Ommen SR, Mital S, Burke MA, Day SM, Deswal A, et al. 2020 AHA/ACC Guideline for the Diagnosis and Treatment of Patients With Hypertrophic Cardiomyopathy: A Report of the American College of Cardiology/American Heart Association Joint Committee on Clinical Practice Guidelines. *Journal of the American College of Cardiology*. 2020;76(25):e159-e240.

2. Humbert M, Kovacs G, Hoeper MM, Badagliacca R, Berger RMF, et al. 2022 ESC/ERS Guidelines for the diagnosis and treatment of pulmonary hypertension. eng. *The European respiratory journal*. 2023;61(1)

3. Heidenreich PA, Bozkurt B, Aguilar D, Allen LA, Byun JJ, et al. 2022 AHA/ACC/HFSA Guideline for the Management of Heart Failure: Executive Summary: A Report of the American College of Cardiology/American Heart Association Joint Committee on Clinical Practice Guidelines. *Circulation*. 2022;145(18):e876-e894.

4. Spyropoulos AC, Anderson FA, FitzGerald G, Decousus H, Pini M, et al. Predictive and associative models to identify hospitalized medical patients at risk for VTE. *Chest*. 2011;140(3):706-714.

5. Petersen S, Peto V, Rayner M. *Coronary heart disease statistics: 2003*. British Heart Foundation; 2003.

6. Williams B, Mancia G, Spiering W, Agabiti Rosei E, Azizi M, et al. 2018 ESC/ESH Guidelines for the management of arterial hypertension: The Task Force for the management of arterial hypertension of the European Society of Cardiology and the European Society of Hypertension: The Task Force for the management of arterial hypertension of the European Society of Cardiology and the European Society of Hypertension. *Journal of Hypertension*. 2018;36(10):1953-2041.

7. Stout KK, Daniels CJ, Aboulhosn JA, Bozkurt B, Broberg CS, et al. 2018 AHA/ACC Guideline for the Management of Adults With Congenital Heart Disease: A Report of the American College of Cardiology/American Heart Association Task Force on Clinical Practice Guidelines. *Circulation*. 2019;139(14):e698-e800.

8. Joglar JA, Chung MK, Armbruster AL, Benjamin EJ, Chyou JY, et al. 2023 ACC/AHA/ACCP/HRS Guideline for the Diagnosis and Management of Atrial Fibrillation: A Report of the American College of Cardiology/American Heart Association Joint Committee on Clinical Practice Guidelines. *Circulation*. 2023;

# Table S1 ICD code of outcome events

| Pulmonary embolism |  |
| --- | --- |
| I26.900x001 | Pulmonary embolism |
| I26.900x002 | Pulmonary thrombosis |
| I26.900x003 | Pulmonary thromboembolism |
| I26.900x005 | Massive pulmonary embolism |
| I26.900x006 | Submassive pulmonary embolism |
| I26.900x010 | Acute pulmonary embolism |
| I26.900x011 | Acute massive pulmonary embolism |
| I26.900x012 | Acute submassive pulmonary embolism |
| I26.900x013 | Acute low risk pulmonary embolism |
| I26.900x014 | Acute pulmonary embolism |
| I26.900x015 | Acute pulmonary thromboembolism |
| I26.901 | Pulmonary thrombosis |
| Deep venous thrombosis |  |
| I80.103 | Iliofemoral vein thrombosis |
| I80.104 | Femoral vein thrombosis |
| I80.203 | Internal iliac vein thrombosis |
| I80.204 | External iliac vein thrombosis |
| I80.206 | Iliac vein thrombosis |
| I80.207 | Deep venous thrombosis of lower extremity |
| I80.208 | Lower extremity deep venous thrombosis |
| I80.209 | Postoperative lower extremity deep venous thrombosis |
| I80.303 | Venous thrombosis lower limb |
| I82.200 | Vena cava embolism and thrombosis |
| I82.200x001 | Vena cava embolism |
| I82.202 | Superior vena cava thrombosis |
| I82.203 | Inferior vena cava thrombosis |
| I82.204 | Inferior vena cava embolism |
| I82.800 | Venous embolism and thrombosis |
| I82.800x003 | Iliac vein embolism |
| I82.800x004 | Internal iliac vein embolism |
| I82.800x005 | External iliac vein embolism |
| I82.800x009 | Axillary vein embolism |
| I82.801 | Internal jugular vein thrombosis |
| I82.802 | Jugular vein thrombosis |
| I82.803 | Subclavian vein thrombosis |
| I82.804 | Axillary vein thrombosis |
| I82.805 | Upper extremity deep venous thrombosis |
| I82.806 | Upper limb venous thrombosis |
| I82.900 | Venous embolism and thrombosis |
| I82.900x001 | Venous embolism |
| I82.900x002 | Venous thrombosis |
| I82.900x004 | Venous thromboembolism |

# Table S2 Number of Patients with Cardiovascular Diseases

| Ward | N (%) |
| --- | --- |
| Coronary heart disease | 17626 (64.72) |
| Hypertension | 16879 (61.98) |
| Atrial fibrillation | 4778 (17.54) |
| Acute coronary syndrome | 2742 (10.07) |
| Cardiomyopathy | 1957 (7.19) |
| Heart failure | 1337 (4.91) |
| Pulmonary hypertension | 974 (3.58) |

# Table S3 Multivariable logistic analysis of risks for hospital-acquired VTE

| Variable | OR | 95% CI | *P*-value |
| --- | --- | --- | --- |
| Coronary heart disease |  |  | <0.001 |
| Chronic coronary heart disease | 1.426 | 0.809-2.513 | 0.220 |
| Acute coronary syndrome | 5.021 | 2.620-9.623 | <0.001 |
| Antiplatelet | 0.289 | 0.173-0.483 | <0.001 |
| Statin | 0.803 | 0.506-1.274 | 0.351 |

CI, confidence interval；OR, odds ratio
